# Visual responses of patients with generalized anxiety disorder who cycling in the virtual sportscapes with different tree cover densities

**DOI:** 10.3389/fpsyt.2022.880586

**Published:** 2022-08-16

**Authors:** Tsai-Chiao Wang, Chia-Liang Tsai, Ta-Wei Tang

**Affiliations:** ^1^Institute of Physical Education, Health and Leisure Studies, National Cheng Kung University, Tainan, Taiwan; ^2^Department of Leisure and Recreation Management, Asia University, Taichung, Taiwan

**Keywords:** tree cover density, virtual sportscapes, pupil size, fixation time, fixation count

## Abstract

The tree density of virtual sportscape is the main factor that determines the benefits that generalized anxiety disorder (GAD) patients can obtain when they exercise with virtual environment. By using pupil size, fixation count and time as metrics, this research aimed to clarify the relationship between tree cover density and stress in the virtual environment. Ninety GAD patients were randomly grouped into the 36–60% tree density (high tree density, HTDS), 20–35% tree density (medium tree density, MTDS), or control groups (*n* = 30). Researchers used eye-tracking technology to analyze fixation time, fixation count and changes in pupil size to evaluate the stress changes of participants after 20 min of aerobic exercise in a virtual environment. The results showed that pupil size expanded in GAD patients after exercising in the virtual environment. Furthermore, GAD patient cycling in the MTDS group can show smaller pupil size than those in HTDS. Those results suggest that GAD patient cycling 20 min in the MTDS group can perceived lower stress. The results of eye tracking analysis showed that GAD patients spend more time and counts observing tree elements in HTDS and MTDS sportscapes. Specifically, they spent more 48% and 27% time on tree and green plants in the HTDS condition and MTDS condition, respectively, than in non-natural sportsscapes. Although 36–60% tree density of virtual natural sportscape can get more visual attention from GAD patients, 20–35% tree density of virtual natural sportscape is more capable of reducing their stress.

## Introduction

Patients with generalized anxiety disorder (GAD) are unwilling to interact with others (1) and avoid outdoor exercise (2), resulting in increasingly poor psychological and physiological health (1, 2). Adults with GAD excessively and persistently worry about many events or activities, causing them to feel immense stress and difficulty in relaxing (3, 4) which even causes daily life to become difficult. Adults with GAD will experience a series of psychological and physiological symptoms, such as feeling tense or fear, tachycardia, tachypnea, and hyperventilation (5). GAD greatly impacts the quality of life of patients (1, 6) and will increase usage of medical services and direct and indirect medical costs (7, 8).

Virtual exercises that promote psychological and physiological health in patients with psychological disorders had attracted the attention of practitioners and researchers. Exercising in a virtual environment solves a key clinical problem: enabling adults with GAD to improve their psychological and psychological health without having to interact with others (2). Virtual reality (VR) is regarded to be a new method for promoting physical activities and has great potential for improving individual health and lifestyle habits (9). In particular, air pollution in cities and even suburbs has roused concern that outdoor exercise may have adverse health effects (10). Studies proved that outdoor exercise in environments with poor air quality may have negative effects on cognitive function (11) and blood pressure (10). Therefore, engaging in virtual sports at home is a good choice to promote health. Furthermore, simulation of a sportscape that enables adults with GAD not to interact with others is a key factor for promoting exercise in these patients. In order to simulate a realistic exercise environment, we examined the contributions of characteristics of a virtual exercise environment (such as tree density) to obtaining visual attention of cycling subjects.

Stress reduction theory is an important framework that explains why contact with nature may promote stress reduction through relaxation of the parasympathetic nervous system (12, 13). This theory states that stress is produced when an individual encounter an event or situation that is unfavorable, threatening, or challenging (14). Several studies have found that the natural environment can induce alpha waves that represent relaxation, and the lower blood flow in the pre-frontal cortex may represent the physiological characteristics of this process (15–17). In addition, people in an urban environment were found to have activated the cingulate gyrus of their brains, which reflects the effort of attention that consumes cognitive resources (16–19).

However, Martens, Gutscher and Bauer (20) found that not all forest environments have the same effect. The reason for this phenomenon is related to tree density in the environment (21). investigated the correlation curve between tree density and physiological stress, and they particularly examined tree density from 1.7 to 62.0%. There is an inverted U-shaped curve between tree density and physiological stress, and the greatest effect of tree density on stress reduction was within 24 to 34%. Similarly, the vegetation density of the community also has a significant effect on reducing anxiety. Higher vegetation density will not lead to a higher anxiety-reducing effect. Moderate vegetation density can achieve the best effect of reducing anxiety (22). Especially, Cox et al. (22) found that the probability of mild or severe depression is significantly decreased when vegetation cover in a neighborhood is 20%.

In certain environments, such as medium depth and complexity, presence of visual focus, and environments with plants and water, attention will be attracted to these environmental elements, which may even block pessimistic thoughts (21). These thoughts are replaced by positive emotions and induce recovery of depressed cognitive behavior and dysregulated physiology (23). The results of many studies based on the stress reduction theory by Ulrich (24) also showed that natural environments (environments with higher restorative potential) can decrease stress-related physiological markers compared with urban environments (environments with lower restorative potential) (18, 19, 21, 25).

Previous studies have confirmed that the natural environment can reduce people's perceived anxiety compared with the built environment [i.e., (26)]. However, Researchers still don't understand the characteristics of the natural environment (for example: how much tree cover density) can promote people's anxiety reduction (27). Tree cover density is considered to be a main characteristic in the virtual environment that will affect the experience and exercise performance of adults during exercise (21, 28). As tree density increases, the view of the sky will be blocked. Excessively high tree density may have different effects on psychological health. For example, Jiang et al. (21) found that speed for reducing stress is slower in adults under high tree density, which may be due to decreased openness under high tree density. Therefore, humans may have similar preference for openness and greenness (29). A place with appropriate tree density may be more suitable for recreational activities than a scenario with high tree density. However, previous studies did not examine the psychological effects of tree density on people who exercise. Based on the study results on vegetation cover by Cox et al. (22) and tree density by Jiang et al. (21), tree density classified into high tree density (36–60%) and medium tree density (20–35%) in this study.

As eye movement tracking technology can obtain objective psychological response data, it has broad application prospects in environmental psychology. Previous psychological recovery studies also encourage the use of eye movement tracking technology to examine the effects of the environment on emotions and cognition (30, 31). When the eye receives different visual stimuli, the pupil will dilate or constrict (32). Accordingly, researchers can record and analyze eye movement data to determine the cognitive experience of the subject in a virtual exercise environment.

People use pupils to perceive external environmental stimuli and pay attention to targets of interest. Several findings confirm that people's viewing natural visual experiences are important in triggering restorative responses (31), however, systematic studies on the characteristics of visual patterns (fixation and pupil size) associated with people's observing of restorative environments are still lacking (25). Previous research used eye movement trackers to investigate the type of eye movement when subjects were asked to assess the possibility of rest and recovery in the presented environment (i.e., (27, 30)). When adults observe a city park image, previous research found that benches, shrubs, and trees are objects with the longest fixation duration, showing that adults are observing objects that are more likely to induce relaxation (i.e., (27, 30)). On the other hand, changes in pupil size can reflect the level of perceived stress in an individual (25, 30). Martínez-Soto et al. (25) employed eye movement tracking technology to determine the differences in pupil size and visual behavior in scenarios with high restorative potential (HRP, such as natural environments) and low restorative potential (LRP, such as cities without natural environments). They found that, relative to low restorative potential environmental perception, restorative environmental observation was associated with decreased eye movement activity (include fixations and pupil dilatation), which may reflect a decrease in cognitive effort when dealing with natural scenes. In the context of restorative sportscapes research, however, there are very few studies that examined the relationship between pupil size and stress.

Individuals may simultaneously prefer openness and greenness (29). A place with appropriate tree density may be more suitable for recreational activities than a scene with high tree density. As tree density increases, the view of the sky will be blocked. High tree density may decrease overall openness, thereby decreasing the stress reduction speed. Jiang et al. (21) investigated the correlation curve between tree density and physiological stress, and they particularly examined tree density from 1.7 to 62.0%. This was an inverted U-shaped curve and tree density that has the greatest effect on stress reduction was from 24 to 34%. Jiang et al. (21) were surprised to find that the inverted U-shape can best describe the data relationship between tree density and physiological stress. This may be because dense vegetation causes discomfort in people. When vegetation is sufficiently dense to obstruct vision, it usually results in discomfort or even fear (33). Under medium tree density sportscapes (MTDS), adults with GAD will feel less stress than high tree density sportscape (HTDS).

In addition, the immersion effects of a virtual environment (34) enable users to experience a forest- or green space-like environment indoors and allows them to exercise in that immersive environment (30). When individuals see images that invoke pleasure, their pupils will dilate (35, 36). When an individual cycle outdoors, he/she will browse the surrounding landscape. While cycling in a natural environment, the adults will be attracted by the surrounding natural environment and continuously sees a green landscape (24, 37). Further, these green landscapes will cause the individuals to relieve stress through visual perception (24). Adults with GAD only need passive observing the surrounding environment and allow natural scenery to be perceived by their eyes during cycling, and they can experience a sense of psychological freedom (38, 39) and then reduce anxiety sensitivity (40, 41). The smaller the pupil size, the higher the value of recovery possibility (25). However, too much dense vegetation will consume more cognitive effort from adults (25). Specially, high-density vegetation cannot produce more peace. The relationship between vegetation density and stress reduction is inverted U-shaped, and stress reduction will weaken as the number of vegetation increases (19, 21). Thus, during aerobic exercise in a virtual natural environment, the relationship between pupil size and stress should be non-linear. Rather, a moderate pupil size can reflect the low-level stress of adults with GAD.

### Objectives and research hypotheses

Current research still does not have a clear understanding of the characteristics of the virtual sportscapes that enables adults with GAD to ride in a low-stress indoor environment. Therefore, the aim of this study was to examine the effect of virtual natural landscapes with high or moderate tree cover density in a cave virtual environment (VE) system on reducing stress in adults with GAD. Combining immerse VR, and eye-tracking, a VR exercise intervention was designed to examine their responses to different tree cover densities (high tree density, HTDS, and medium tree density, MTDS) in natural sportscape. In addition to physiological measurements of stress reaction, visual attention was also measured. The hypotheses of this study were that cycling in a VE with medium tree density will lower (1) pupil size, (2) increase fixation count and (3) fixation time comparably when cycling in a VE with high tree cover density compared with medium tree cover density. In order to validate the proposed hypotheses, a randomized controlled trial was conducted.

## Methods

**Figure 1:**
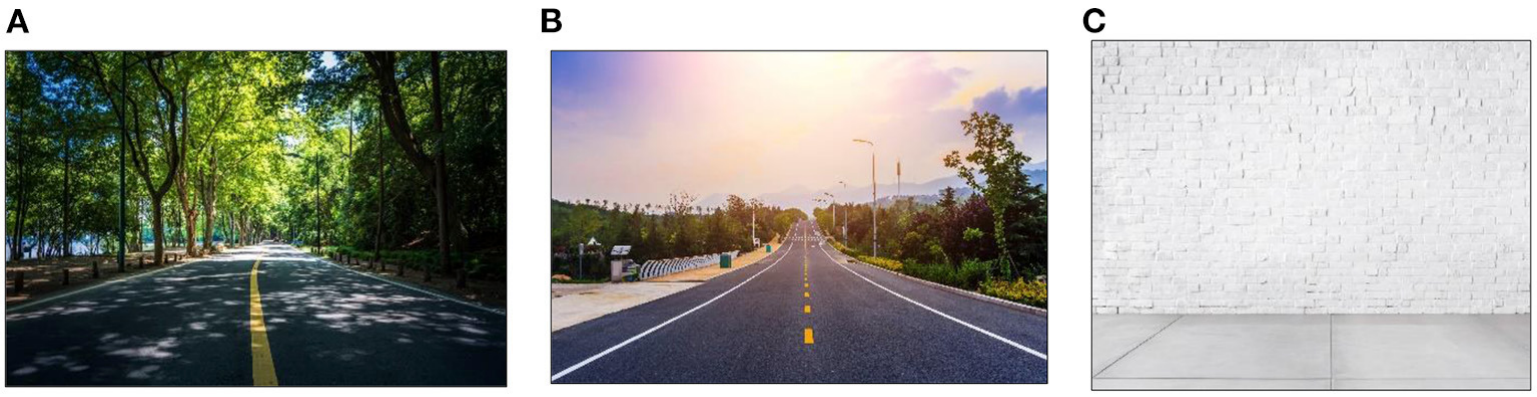
Experimental images. **(A)** high tree density sportscape (HTDS); **(B)** medium tree density sportscape (MTDS); **(C)** control condition. (Experimental images from evening_tao / Freepik).

To examine the effects of tree density in sportscapes on visual and stress in adults with GAD, we recruited adults with GAD as participants. This study was performed at National Cheng Kung University, Taiwan. We constructed 2 types of virtual sportscapes and 1 control condition for the experiment (Figure 1). We asked these participants to cycle at different tree densities and recorded their pupil size, fixation count and fixation time using the eye-tracking equipment.

### Participants

The authors attended an older adult's meeting in Tainan, Taiwan, and invited older adult with GAD tendencies to fill out the GAD-7 scale first. The authors formally invited each older adult to join the study after physicians assessed that the older adult met the criteria for GAD. Participants were selected for this study based on the following inclusion criteria: (a) People between the ages of 50 and 75 have relatively more stress and may get more benefits from physical activity. People aged 50–59 (compared to ≥80 years) have a higher rate of GAD (42), and the risk of anxiety decreases by 40% after 75 years of age, and 47% after 80 years of age (43). Thus, age between 50 and 75 years as exercise has many benefits for middle-aged and elderly individuals (such as decreasing mortality rate, delaying cognitive aging, and decreasing medical costs) (44, 45); (b) A score above 5 on the GAD-7 scale, representing participants have at least mild anxiety symptoms; (b) Normal body mass index (BMI) defined by the Taiwan Ministry of Health and Welfare based on relevant disease incidence data and risk of death in Asian populations (18.5 ≤ BMI < 24 kg/m2) (46). The exclusion criteria are participants with the following: (a) obsessive compulsive disorder or other anxiety disorder; (b) a mini mental state examination (MMSE) score of <24, representing cognitive impairment; and (c) claustrophobia, as this experiment was performed in an indoor immersive surrounding sound system. Ninety eight participants were contacted and 8 participants were excluded as they suffer from other psychiatric disorders or were unable to complete the entire experiment due to physical reasons.

Power analysis (G^*^Power 3.1.9.4) was used to calculate the sample size required to obtain at least small-to-medium results (*r* = 0.20) at an alpha of 0.05 (two-tailed) (47). Power was set as 0.80 (48). G^*^Power analysis showed that the sample size required was *N* = 54. Therefore, the sample size used in this study (*n* = 90) conforms to the test hypotheses.

### Ethical consideration

Participants participated in this study entirely voluntarily. All participants had to read the instructions and provide informed consent before starting the investigation according to the Declaration of Helsinki. The researchers informed the participants that they could discontinue the investigation at any time for any reason. This study was approved by the institutional review board of National Cheng Kung University (B-ER-107-150). The confidentiality of personal data was protected under the Taiwan Data Protection Law.

### Experimental procedure

This experiment requires participants to focus their attention for around 1 H. To decrease the potential risk of interfering psychological and physiological responses and complicating factors, the researchers called the subjects with GAD 24 h before the experiment to remind them to abstain from certain behaviors (such as pulling all-nighters, drinking caffeinated beverages, and taking medications).

Ninety adults with GAD were randomly grouped into the high tree density sportscape (HTDS, *n* = 30), medium tree density sportscape (MTDS, *n* = 30), or control groups (non-virtual environment, *n* = 30). Every participant was required to arrive at the laboratory at 8: 30–9:30 a.m. to control for the effects of the circadian rhythm. After the participant has arrived at the laboratory, the research assistant explained the experimental procedure and requested that the participant completed the informed consent form, demographic survey form, and MMSE and GAD-7 questionnaires. In addition, their height and weight were measured to calculate the BMI. Following that, eye movements, and heart rate (HR) were measured to ensure that there is no difference in relaxation status and emotional status before the experimental intervention.

**Figure 2:**
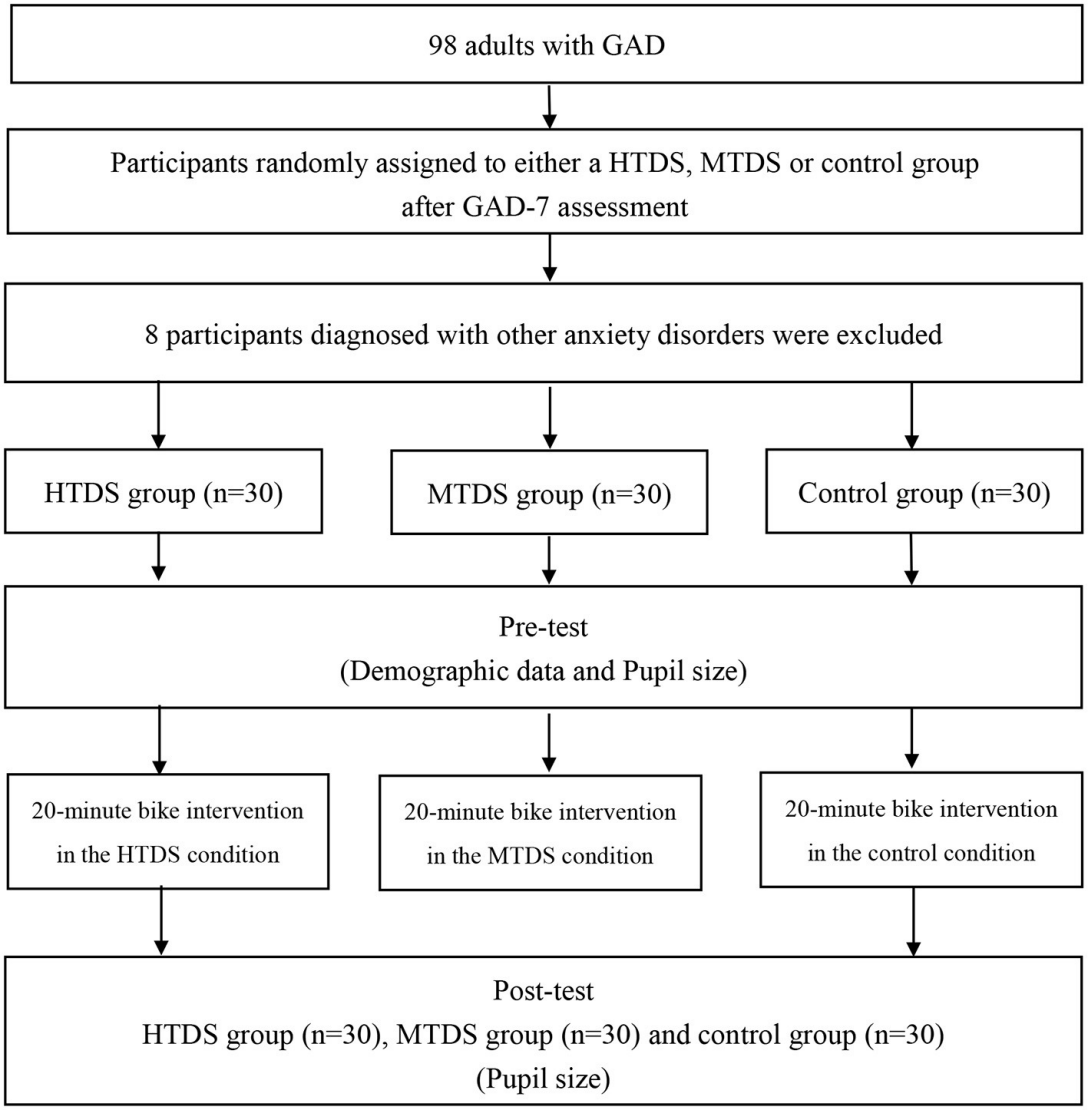
Flowchart of the study.

Participants from the HTDS and MTDS groups cycled for 20 min in Cave VE (Figure 1). Participants wore the Polar optical HR sensor on their wrists to monitor their HR during cycling. All participants were required to carry out moderate intensity exercise (50–60% HRmax). In the HTDS and MTDS groups, machine-simulated forests, parks, trees, rivers, and other landscapes were projected in Cave and moved as the participant cycled. In the HTDS, trees accounted for 36–60% of the entire sportscape. In the MTDS, trees accounted for 20–35% of the entire sportscape. After exercise intervention, HR, and eye movements were measured (see Figure 2).

### Exercise duration

With regards to the duration of exercise required to have positive psychological effects, existing papers mostly found that longer exercise duration such as 20 min or more seems to have the greatest psychological benefit (49). Therefore, this study adopted a 20 min cycling exercise as the duration for a single exercise intervention.

### Virtual environment

The researcher constructed a CAVE VE around the participants so that the participants can exercise in that surround environment. The Cave VE system was installed in a dimly-lit soundproof room and mainly projects the sportscape around the participant to enhance immersion and presence so that the participants felt that they were cycling outdoor (34, 50). The VE was presented in images within the visual field of the participants. The VE surround screens were at the front, left, right, and bottom of the participants. The bicycle was 2 meters away from the front screen. Several screens surround the participants to create a surround scenario. Surround 3D projection technology was used to project images on the screen to create a complete surround experimental scenario for the participants (26, 34). The CAVE virtual reality system hardware consists mainly of projectors and screens. For surround VR projection, 2 projectors were used to project images at 270 degrees and participants can view the cycling track images used in the 3D experiment with the naked eye. This system employs wireless streaming technique so that the participants' cycling speed and the image movement speed are synchronized in the VE (combined virtual reality equipment, Taiwan patent no. I67522). Virtual reality systems similar to Cave were successfully used in previous studies (26, 50)]. All experimental steps were performed in the VE laboratory of National Cheng Kung University. During the experiment, temperature was controlled at 24–25°C and relative humidity was controlled at 50–60%.

### Tree density calculations

**Figure 3:**
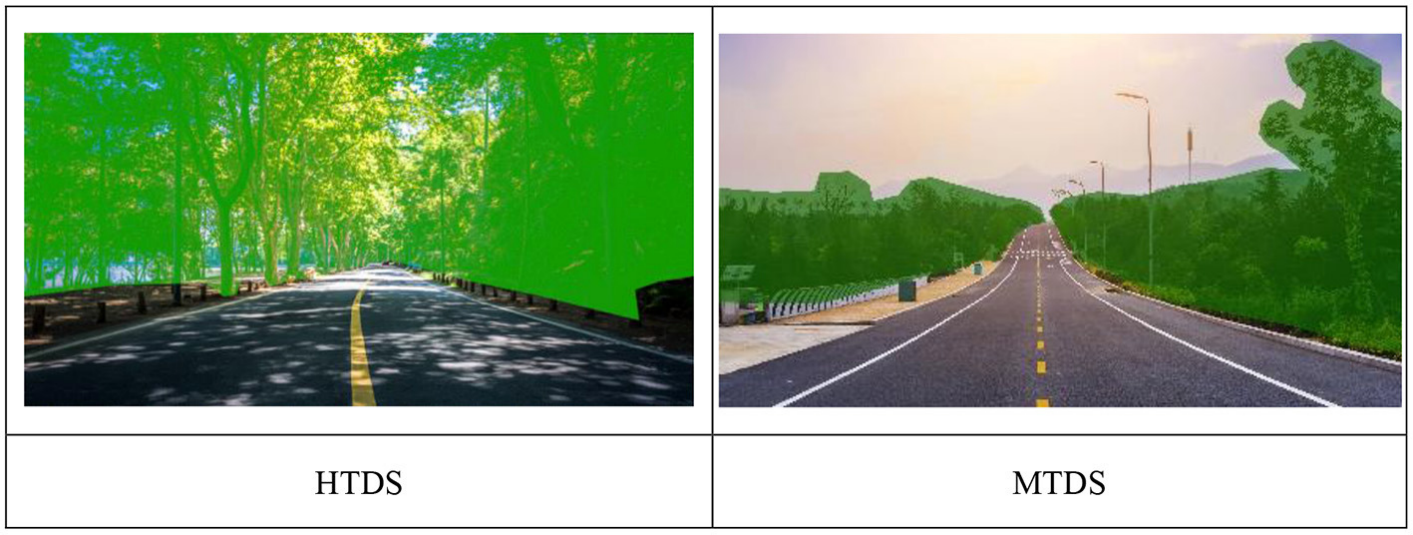
Example images from the HTDS and MTDS that were shown to participants through cave virtual environment.

A 20 min virtual cycling track was employed and images were extracted once every 2 min. Ten images each were extracted in the high and medium tree density environments. Following that, the researcher calculated the tree density of each image. The recommendations of Jiang et al. (21) were used to measure tree density using the Adobe Photoshop software to calculate the total pixels in green areas on trees, which was used to calculate the pixel percentage of trees in the entire photograph. The images were classified as high tree density (tree density accounted for 36–60% of the entire image) and medium tree density (tree density accounted for 20–35% of the entire image) (51) (see Figure 3).

### Instruments

The Tobii T/X eye tracker (Tobii G2; Tobii Pro Glasses2-50, Danderyd, Sweden) was used to track the eye movement of participants. The data (include the fixation counts, fixation time and pupil dimension) were collected using specialized eye movement analysis software (Tobii Studio).

The initial diagnosis of GAD based on the generalized anxiety disorder 7-item (GAD-7) scale as this has high sensitivity and specificity for GAD detection (52, 53). GAD-7 is a reliable self-report measure with high internal consistency and good test-retest reliability (54–57).

Participants were asked to rate how they felt in their lives: “In the past 2 weeks, how often have you been bothered by the following 7 items?” Each item was measured on a four-point Likert scale (0 = not at all sure, 1 = several days, 2 = over half the days, and 3 = nearly every day). The total score (ranges from 0 to 21) is divided into four different levels of severity: asymptomatic (0 to 4); mild anxiety symptoms (5 to 9); moderate anxiety symptoms (10 to 14) or severe anxiety symptoms (15 to 21). Older adults with a score of 5 or higher were selected for this study. A higher score means a higher severity.

### Visual attention

To measure visual attention, the authors divided the sportscapes image into 2 areas of interest (AOI), including 1. trees, and 2. road. This method is used to confirm which part of the sportscapes image the observer is most interested in. Eye tracking technology visualizes visual attention in the form of a gaze plot, allowing researchers to explore visual attention. The key indicators used to assess individual's visual attention were the average fixation count and the total fixation time (58). The average fixation count is the number of times that the individual interacts with the stimuli, with higher total fixation counts indicating that individuals felt the observed stimulus more attractive to them (26, 59). The total fixation time is the processing time when the individual to observe the stimuli, with longer total fixation time implying that individuals spent more time exploring the information or the relationships between the internal and external representations (59, 60). The phenomenon suggests that the individual's visual attention was more attracted during that time (59, 61). Previous neuroscience visuo-cognitive research usually uses a threshold of 200 ms (62, 63). Therefore, values below 200 ms were excluded from subsequent analyses (64).

### Pupil size

The definition of pupil size in this study was based on the actual external physical dimensions of the pupils. Pupils will constrict or dilate with light, illumination, and stimulation and pupil diameter ranges from 1.3 to 10 mm (65, 66). Pupil size is considered a reliable parameter for identifying an individual's stress state (67). The amount of pupil diameter decreases to represent stress reduction and emotional relaxation (67). This study used the amount of change in pupil size as an indicator of stress.

### Data analysis

All statistical analysis was carried out using SPSS 21.0 (SPSS Inc, IBM Chicago, IL, USA). Descriptive statistical data were expressed as mean ± SD (Table 1). One way ANOVA was used for inter-group comparison of demographic data. Values of pupil size, fixation time and fixation count were used for a 3 (groups: HTDS, MTDS, and control) × 3 (time points: before intervention, during intervention, and after intervention) one way ANOVA. Bonferroni correction was used for paired multiple posterior comparisons of mean values to determine if there are significant differences. A difference of *p* < 0.05 was considered to be statistically significant. Cohen's d was used for estimation of effect size for significant *t*-test results (68) and effect sizes were classified as low, medium, and high based on values of 0–0.2 m 0.2–0.5, and 0.5–0.8, respectively (48).

**Table 1:** Baseline demographic characteristics of the participants [mean (SD)].

**Variables**	**HTDS group (*n* = 30)**	**MTDS group (*n* = 30)**	**Control group (*n* = 30)**	***p*-value**
Age (year)	58.43 (7.37)	59.87 (6.99)	60.85	0.25
Gender (M/F)	13/17	14/16	14/16	0.47
Height (m)	1.60 (0.08)	1.60 (0.09)	1.62 (0.09)	0.91
Weight (kg)	60.22 (10.93)	61.97 (13.28)	61.11 (12.78)	0.53
BMI (kg/m^2^)	21.54 (1.25)	21.56 (1.77)	21.82 (1.34)	0.88
GAD levels (Medium/low)	18/12	18/12	17/13	0.47
MMSE (score)	28.81 (1.44)	29.13 (1.11)	28.81 (1.44)	0.29
GAD-7 (score)	12.43 (2.73)	12.73 (3.52)	12.43 (2.73)	0.78
Resting HR (count/minute)	77.91 (6.84)	79.83 (6.60)	78.17 (6.65)	0.46

SD, standard deviation; BMI, body mass index; GAD, generalized anxiety disorder; MMSE, mini-mental state examination; HR, heart rate; HTDS, high tree density sportscape; MTDS, Medium tree density sportscape; HR, heart rate.

## Results

### Demographic characteristics

No participant reported any discomfort throughout the 20 min experiment. There were no significant differences in demographic variables between the HTDS, MTDS and control groups before the intervention (Table 1). In this study, 41 men and 39 women were included. The overall mean age was 59.86 ± 7.46 years.

### Pupil size

**Figure 4:**
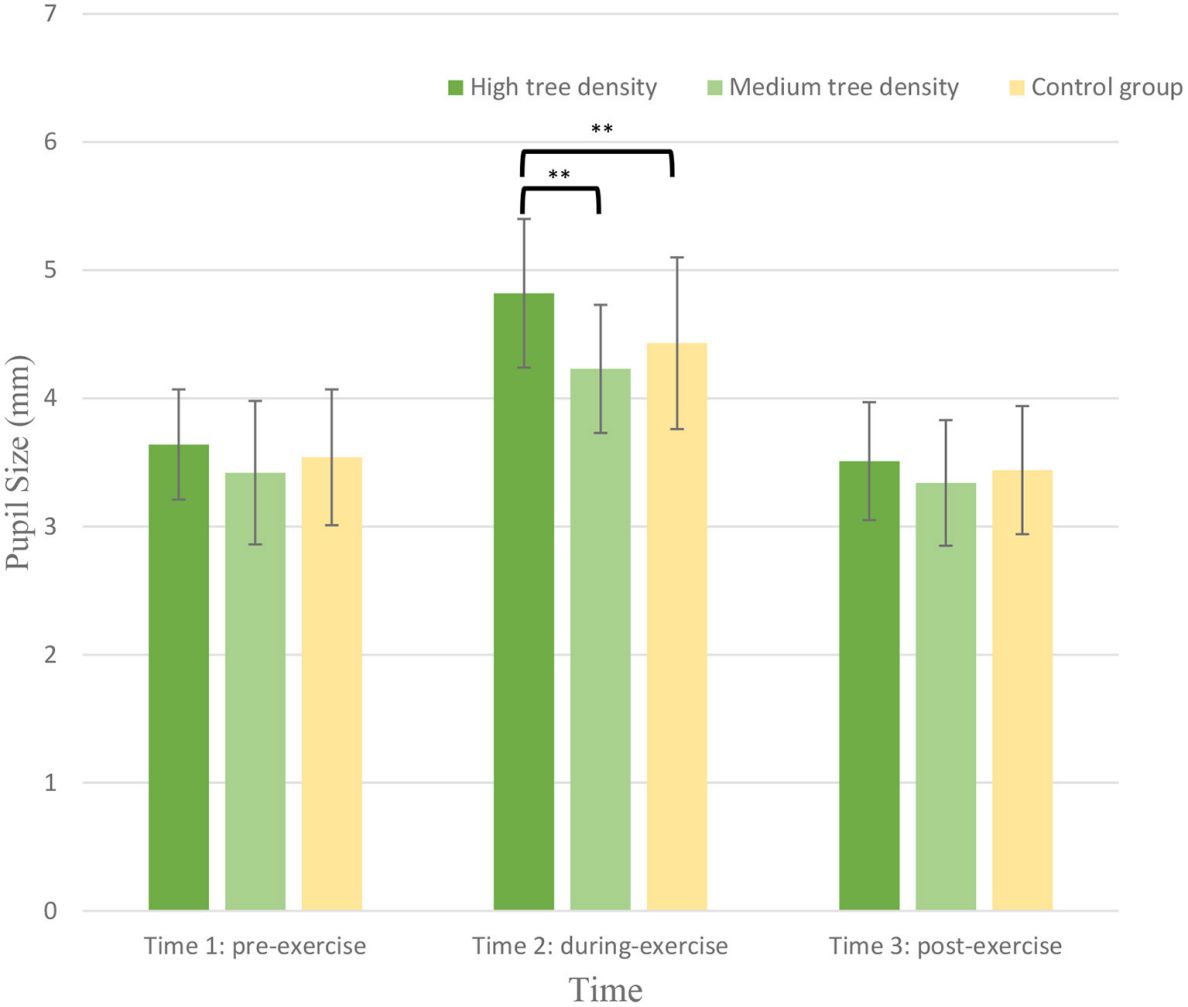
Pupil size for the high tree density sportscape (HTDS) and the medium tree density sportscape (MTDS) groups at 3 different time points. (**p < 0.01).

**Figure 5:**
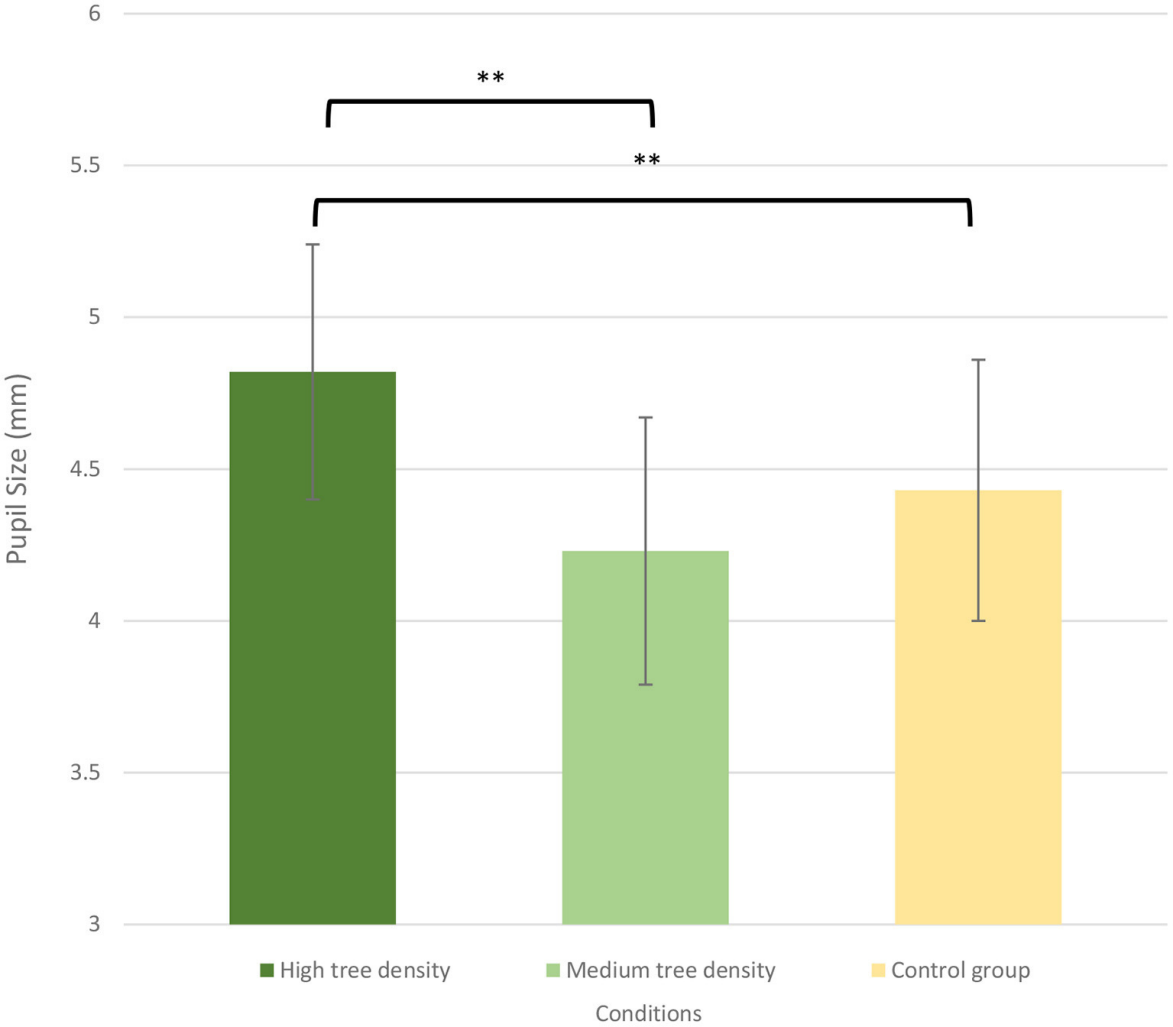
Pupil size for the high tree density sportscape (HTDS) and the medium tree density sportscape (MTDS) groups during exercise. (**p < 0.01).

As shown in Figure 4, the RM ANOVA on the pupil size revealed a significant main effect of *Time* [*F*_(2,89)_ = 128.36, *p* = < 0.001, $\eta_{p}^{2}$ = 0.30] and *Group* [*F*_(2,89)_ = 3.34, *p* < 0.01, $\eta_{p}^{2}$ = 0.15], showing that the during-exercise pupil size (4.28 ± 0.08) were higher than the pre-exercise values (3.54 ± 0.07) and post-exercise (3.43 ± 0.07) across the three groups, and the pupil size for the HTDS group (3.97 ± 0.09) were higher than those for the MTDS group (3.71 ± 0.10) and control group (3.57 ± 0.15) across the three time points (See Figure 4). The main effect was superseded by the *Time* × *Group* [*F*_(4,89)_ = 16.94, *p* < 0.001, $\eta_{p}^{2}$ = 0.15] interaction. The *post-hoc* analyses indicated that the during-exercise pupil size were lower than the pre-exercise values and post-exercise values for the MTDS (pre-exercise vs. during-exercise vs. post-exercise: 4.82 ± 0.58 mm vs. 4.13 ± 0.5 mm vs. 4.43 ± 0.67 mm; *p* < 0.01) (See Figure 5), showing that pupil size in the MTDS group were smaller than those in the HTDS and control group. Thus, the hypothesis 1 was supported.

### Average total fixation count and fixation time

**Figure 6:**
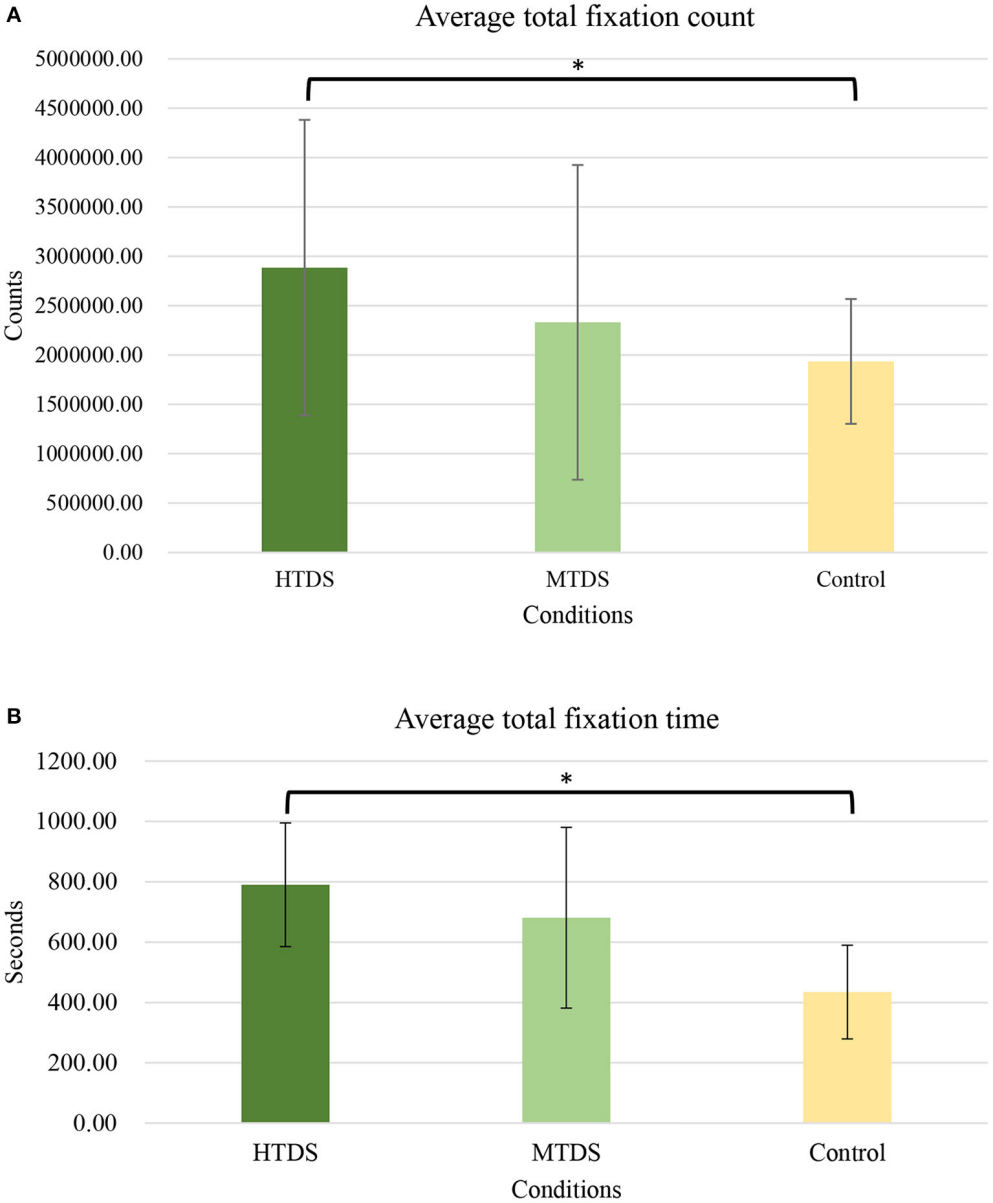
**(A)** Average total fixation count for the high tree density sportscape (HTDS) and the medium tree density sportscape (MTDS) groups during exercise. (*p < 0.05). **(B)** Average total fixation time for the high tree density sportscape (HTDS) and the medium tree density sportscape (MTDS) groups during exercise. (*p < 0.05).

As shown in Figures 6A,B, a one-way ANOVA analysis showed significant differences in total fixation count (*F*_(2, 87)_ = 4.8, *p* = 0.02) and total fixation time (*F*_(2, 87)_ = 5.32, *p* = 0.01) across the three groups during cycling. *Post-hoc* pairwise comparisons showed that the HTDS group's total fixation count was significantly higher than the MTDS group (2885284.75 ± 1497042.52 vs. 2331572.33 ± 1593762.72, *p* = 0.67) and control group (2885284.75 ± 1497042.52 vs. 1935592.6 ± 632001.02, *p* = 0.01). *Post-hoc* pairwise comparisons also showed that the HTDS group's total fixation time was significantly higher than the MTDS group (790.22 ± 204.97 vs. 680.89 ± 299.46, *p* = 0.43) and control group (790.22 ± 204.97 vs. 434.48 ± 155.32, *p* = 0.01), showing that the HTDS group have higher visual attention (average total fixation count and the total fixation time) compared to the control group. There is no significant difference between MTDS and Control group for average total fixation count and fixation time. The hypotheses 2 and 3 were supported.

## Discussion

This study examined the effects of different tree densities on visual behavior in adults with GAD and found that the tree density of simulated sportscape is a key factor that affects their exercise experience. Pupil diameter was the largest when adults with GAD exercise in a high tree density environment, followed by control group. In contrast, pupil dilation was the lowest in adults with GAD who exercised in the medium tree density group. Those results imply that exercising under medium tree density can best lower stress.

This research contributes to the research on promoting adults with GAD to exercise at home has the following contributions in the following ways: First, the tree density of the virtual sportscape is a key factor that determines the degree of stress during exercise. Shanahan et al. (69) argued that the interaction between the natural environment and physical activity can enhance the benefits of physical activity. The results of this study further point out that not all exercises in the natural environment can obtain the same benefits, but depend on the tree density. Compared with a high tree density environment, exercise in a medium tree density environment can bring lower stress to adults with GAD. Thus, exercise with VE could be a suitable stress reduction technique in adults with GAD regardless of the environment introduced (2). Therefore, VE with a medium tree density sportscapes can help adults with GAD who are afraid of interacting with others or the outside world to be not feel stressed, and thus willing to ride bicycles with VE in their homes.

Previous studies on the relationship between tree density and stress recovery also found similar results. For example, Jiang et al. (21) examined the tree density of urban landscapes and found that observed recovery is decreased (self-reported stress recovery) when tree density in the residential area exceeds the medium level (34–62% of the visual field). Even though they did not specifically measure perceived safety but they deduced that “when tree density is sufficient to block the line of sight”, it usually leads to discomfort or even fear [(21). p. 34]. These findings mean that perceived security may have a mediating role in the relationship between the closeness of green space and perceived recovery. Therefore, perceived negative emotions and perceived lack of security may be one of the causes of lack of relaxation when adults with GAD perform aerobic exercise in a high tree density setting.

Second, in the advertisement image, the type of servicescape may affect the eye movement pattern of the individual extracting information from it. The data of eye movement analysis shows that there are fewer fixations in the homogeneous servicescape. This result means that, compared with the diversified servicescape, the homogeneous servicescape has simple characteristics (25) and does not need to be explored intensively (70). Furthermore, the longer fixation time of an object is considered to represent more interest or liking (71, 72) and more likely to induce emotional arousal (73). The data of eye tracking analysis shows that HTDS will have more fixation time than MTDS. This result implies that the individual is more interested in HTDS and perceives higher stress. This result is consistent with the previous description of the affective valence of HRP and LRP photos (25, 74).

Third, the high tree density of virtual sportscapes will over-attract the visual attention (pupil size becomes larger) of adults with GAD during exercise. The ANOVA results of the relationship between pupil size and tree density show that HTDS makes the pupil size of adults with GAD larger than the pupil size of their cycling under MTDS, and control conditions. Also, the pupil size of adults with GAD cycling in a medium density sportscapes was smaller than that of a high density sportscapes and the control group. This may be because adults with GAD cycling in HTDS will increase the visual attention to the surrounding environment and then cause pupil dilation.

Gavas et al. (75) found that the same pupil dilation may represent different meanings, depending on the situation. For example, pupil dilation will be greater if the natural environment is more interesting or invokes negative emotions. Too dense forest might cause adults with GAD to be unable to obtain the benefits of relaxation. High tree density (36–60%) will cause people who exercise to pay more attention to search for potential dangers (76, 77) or even feel frightened (21, 78). Therefore, their pupils will be more dilated, which decreases the level of relaxation. These results may mean that medium tree density can cause people who exercise to feel the least stressful and do not invoke negative emotions (such as fear and terror) due to overly dense tree landscapes, which consumes their visual attention (lead to pupil dilation).

### Research limitations and future research suggestions

There were some limitations in this study. Due to the limitations of current machine learning techniques, tree coverage density of virtual sportscapes cannot be evaluated automatically. Method development with this capability is needed to advance the virtual sportscapes field in the future. Furthermore, the relationship between tree cover density and pupil size may be a complex curve relationship and future studies can further examine the correlation between tree density and pupil size.

## Conclusion

The results of this study found that the green coverage rate in the sportscapes can affect the visual and psychological responses of adults with GAD. Especially, 36–60% tree density of virtual natural sportscape can get more visual attention from adults with GAD, 20–35% tree density of virtual natural sportscape can reduce their stress more.

To meet the needs of adults with GAD to engage in physical activity indoors. Virtual sports equipment companies can design virtual sportscapes with different functions for GAD to choose. For example: design a sportscape with 20–35% tree coverage to meet the needs of adults with GAD who want to relax through exercise. In addition, a sportscape with 36–60% tree coverage can also be constructed, so that adults with GAD can feel interesting and not boring during exercise, and promote the formation of their long-term physical activity habits.

## Data availability statement

The datasets presented in this article are not readily available because the author does not own the datasets. Requests to access the datasets should be directed to Chen, fongjhihchen@gmail.com.

## Ethics statement

The studies involving human participants were reviewed and approved by National Cheng Kung University. The patients/participants provided their written informed consent to participate in this study.

## Author contributions

T-CW and T-WT wrote the first draft. C-LT collected and analyzed the data. All authors contributed to the article and approved the submitted version.

## Conflict of interest

The authors declare that the research was conducted in the absence of any commercial or financial relationships that could be construed as a potential conflict of interest.

## Publisher's note

All claims expressed in this article are solely those of the authors and do not necessarily represent those of their affiliated organizations, or those of the publisher, the editors and the reviewers. Any product that may be evaluated in this article, or claim that may be made by its manufacturer, is not guaranteed or endorsed by the publisher.

## References

[1] Shin KE Newman MG. Self-and other-perceptions of interpersonal problems: Effects of generalized anxiety, social anxiety, and depression. J Anxiety Disord. (2019) 65:1–10. 10.1016/j.janxdis.2019.04.005 31054457 PMC6658327

[2] Wang TC Sit CHP Tang TW Tsai CL. Psychological and physiological responses in patients with generalized anxiety disorder: the use of acute exercise and virtual reality environment. Int J Environ Res Public Health. (2020) 17:4855. 10.3390/ijerph17134855 32640554 PMC7370051

[3] Robichaud M Koerner N Dugas MJ. Cognitive Behavioral Treatment for Generalized Anxiety Disorder: From Science to Practice. New York, NY: Routledge (2019). 10.4324/9781315709741

[4] Tacchini G Vismara M. (2019). Generalized anxiety disorder, somatization, and emotional dysregulation: a possible link. In: Altamura A, Brambilla P, editors. Clinical Cases in Psychiatry: Integrating Translational Neuroscience Approaches. Cham: Springer. 10.1007/978-3-319-91557-9_12

[5] Craske MG Rauch SL Ursano R Prenoveau J Pine DS Zinbarg RE. What is an anxiety disorder?. Focus. (2011) 9:369–88. 10.1176/foc.9.3.foc369 19957279

[6] Higgins C Chambers JA Major K and Durham RC. Healthcare costs and quality of life associated with the long-term outcome of anxiety disorders. Anxiety Stress Coping. (2020) 34:228–41. 10.1080/10615806.2020.1839731 33108887

[7] Hohls JK Koenig HH Raynik YI Hajek A. A systematic review of the association of anxiety with health care utilization and costs in people aged 65 years and older. J Affect Disord. (2018) 232:163–76. 10.1016/j.jad.2018.02.011 29494900

[8] Konnopka A König H. Economic burden of anxiety disorders: a systematic review and meta-analysis. Pharmacoeconomics. (2020) 38:25–37. 10.1007/s40273-019-00849-7 31646432

[9] Wang TC Tsai CL Tang TW Wang WL Lee KT. The effect of cycling through a projection-based virtual environment system on generalized anxiety disorder. J Clini Med. (2019) 8:973. 10.3390/jcm8070973 31277466 PMC6678108

[10] Kubesch NJ De Nazelle A Westerdahl D Martinez D Carrasco-Turigas G Bouso L. Respiratory and inflammatory responses to short-term exposure to traffic-related air pollution with and without moderate physical activity. Occup Environ Med. (2015) 72:284–93. 10.1136/oemed-2014-102106 25475111

[11] Bos I De Boever P Panis LI Meeusen R. Physical activity, air pollution and the brain. Sports Med. (2014) 44:1505–18. 10.1007/s40279-014-0222-6 25119155

[12] Bratman GN Anderson CB Berman MG Cochran B De Vries S Flanders J. Nature and mental health: an ecosystem service perspective. Sci Adv. (2019) 5:eaax0903. 10.1126/sciadv.aax0903 31355340 PMC6656547

[13] Whitburn J Linklater WL Milfont TL. Exposure to urban nature and tree planting are related to pro-environmental behavior via connection to nature, the use of nature for psychological restoration, and environmental attitudes. Environ Behav. (2019) 51:787–810. 10.1177/0013916517751009

[14] Ulrich RS Simons RF Losito BD Fiorito E Miles MA Zelson M. Stress recovery during exposure to natural and urban environments. J Environ Psychol. (1991) 11:201–30. 10.1016/S0272-4944(05)80184-7

[15] Chiang YC Li D Jane HA (2017). Wild or tended nature? The effects of landscape location and vegetation density on physiological and psychological responses. Landsc Urban Plan. 167:72–83. 10.1016/j.landurbplan.2017.06.001

[16] Martínez-Soto J Gonzales-Santos L Pasaye E Barrios FA. Exploration of neural correlates of restorative environment exposure through functional magnetic resonance. Intell Build Int. (2013) 5:10–28. 10.1080/17508975.2013.807765

[17] Tang IC Tsai YP Lin YJ Chen JH Hsieh CH Hung SH. Using functional Magnetic Resonance Imaging (fMRI) to analyze brain region activity when viewing landscapes. Landsc Urban Plan. (2017) 162:137–44. 10.1016/j.landurbplan.2017.02.007

[18] Jiang B He J Chen J Larsen L Wang H. Perceived green at speed: a simulated driving experiment raises new questions for attention restoration theory and stress reduction theory. Environ Behav. (2020) 53:296–335. 10.1177/0013916520947111

[19] Jiang B He J Chen J Larsen L. Moderate is optimal: a simulated driving experiment reveals freeway landscape matters for driving performance. Urban For Urban Green. (2021) 58:126976. 10.1016/j.ufug.2021.126976

[20] Martens D Gutscher H Bauer N. Walking in “wild” and “tended” urban forests: the impact on psychological well-being. J Environ Psychol. (2011) 31:36–44. 10.1016/j.jenvp.2010.11.001

[21] Jiang B Chang CY Sullivan WC. A dose of nature: tree cover, stress reduction, and gender differences. Landsc Urban Plan. (2014) 132:26–36. 10.1016/j.landurbplan.2014.08.005

[22] Cox DT Shanahan DF Hudson HL Plummer KE Siriwardena GM Fuller RA. Doses of neighborhood nature: the benefits for mental health of living with nature. Bioscience. (2017) 67:147–55. 10.1093/biosci/biw173

[23] Norwood MF Lakhani A Maujean A Zeeman H Creux O Kendall E. Brain activity, underlying mood and the environment: A systematic review. J Environ Psychol. (2019) 65:101321. 10.1016/j.jenvp.2019.10132133197760

[24] Ulrich RS. Aesthetic and affective response to natural environment. In: Altman I, Wohlwill JF, editors. Behavior and the Natural Environment. Boston, MA: Springer (1983). p. 85–125. 10.1007/978-1-4613-3539-9_4

[25] Martínez-Soto J de la Fuente Suárez LA Gonzáles-Santos L Barrios FA. Observation of environments with different restorative potential results in differences in eye patron movements and pupillary size. IBRO Rep. (2019) 7:52–8. 10.1016/j.ibror.2019.07.1722 31453409 PMC6704250

[26] Wang TC Cheng JS Shih HY Tsai CL Tang TW Tseng ML. Environmental sustainability on the tourist hotels' images development. Sustainability. (2019) 11:2378. 10.3390/su11082378

[27] Nordh H Hagerhall CM Holmqvist K. Exploring view pattern and analysing pupil size as a measure of restorative qualities in park photos. In II International Conference on Landscape and Urban Horticulture. (2010). p. 767–72. 10.17660/ActaHortic.2010.881.126

[28] Truong MX Prévot AC Clayton S. Gamers like it green: the significance of vegetation in online gaming. Ecopsychology. (2018) 10:1–13. 10.1089/eco.2017.0037

[29] Brown RD Corry RC. Evidence-based landscape architecture: the maturing of a profession. Landsc Urban Plan. (2011) 100:327–9. 10.1016/j.landurbplan.2011.01.017

[30] Nordh H Hagerhall CM Holmqvist K. Tracking restorative components: patterns in eye movements as a consequence of a restorative rating task. Landsc Res. (2013) 38:101–16. 10.1080/01426397.2012.691468

[31] Stevenson MP Dewhurst R Schilhab T Bentsen P. Cognitive restoration in children following exposure to nature: evidence from the attention network task and mobile eye tracking. Front Psychol. (2019) 10:42. 10.3389/fpsyg.2019.00042 30804825 PMC6370667

[32] Engelke U Le Callet P. Perceived interest and overt visual attention in natural images. Signal Process Image Commun. (2015) 39:386–404. 10.1016/j.image.2015.03.004

[33] Jansson M Fors H Lindgren T Wiström B. Perceived personal safety in relation to urban woodland vegetation–a review. Urban For Urban Green. (2013) 12:127–33. 10.1016/j.ufug.2013.01.005

[34] Gomez DH Bagley JR Bolter N Kern M Lee CM. Metabolic cost and exercise intensity during active virtual reality gaming. Games Health J. (2018) 7:310–6. 10.1089/g4h.2018.0012 30325233

[35] Bradley MM Miccoli L Escrig MA Lang PJ. The pupil as a measure of emotional arousal and autonomic activation. Psychophysiology. (2008) 45:602–7. 10.1111/j.1469-8986.2008.00654.x 18282202 PMC3612940

[36] Henderson RR Bradley MM Lang PJ. Emotional imagery and pupil diameter. Psychophysiology. (2018) 55:e13050. 10.1111/psyp.13050 29266253 PMC5940515

[37] Loureiro A Veloso S. Green exercise, health and well-being. In: Fleury-Bahi G, Pol E, Navarro O, editors. Handbook of Environmental Psychology and Quality of Life Research. Cham: Springer (2017). p. 149–69. 10.1007/978-3-319-31416-7_8

[38] Stubbs B Vancampfort D Rosenbaum S Firth J Cosco T Veronese N. An examination of the anxiolytic effects of exercise for people with anxiety and stress-related disorders: a meta-analysis. Psychiatry Res. (2017) 249:102–8. 10.1016/j.psychres.2016.12.020 28088704

[39] Vancampfort D Stubbs B Venigalla SK Probst M. Adopting and maintaining physical activity behaviours in people with severe mental illness: the importance of autonomous motivation. Prevent Med. (2015) 81:216–20. 10.1016/j.ypmed.2015.09.006 26386141

[40] Newman MG Llera SJ. A novel theory of experiential avoidance in generalized anxiety disorder: A review and synthesis of research supporting a contrast avoidance model of worry. Clin Psychol Rev. (2011) 31:371–82. 10.1016/j.cpr.2011.01.008 21334285 PMC3073849

[41] Smits JA Berry AC Rosenfield D Powers MB Behar E Otto MW. Reducing anxiety sensitivity with exercise. Depress Anxiety. (2008) 25:689–99. 10.1002/da.20411 18729145

[42] McDowell CP Dishman RK Vancampfort D Hallgren M Stubbs B MacDonncha C. Physical activity and generalized anxiety disorder: results from The Irish Longitudinal Study on Ageing (TILDA). Int J Epidemiol. (2018) 47:1443–53. 10.1093/ije/dyy141 29982489

[43] Canuto A Weber K Baertschi M Andreas S Volkert J Dehoust MC. Anxiety disorders in old age: psychiatric comorbidities, quality of life, and prevalence according to age, gender, and country. Am J Geriatr Psychiatry. (2018) 26:174–85. 10.1016/j.jagp.2017.08.015 29031568

[44] Samitz G Egger M Zwahlen M. Domains of physical activity and all-cause mortality: systematic review and dose–response meta-analysis of cohort studies. Int J Epidemiol. (2011) 40:1382–400. 10.1093/ije/dyr112 22039197

[45] Tsai CL Wang WL. Exercise-mode-related changes in task-switching performance in the elderly. Front Behav Neurosci. (2015) 9:56. 10.3389/fnbeh.2015.00056 25798097 PMC4351633

[46] Tsai CL Pan CY Chen FC Huang TH Tsai MC Chuang CY. Differences in neurocognitive performance and metabolic and inflammatory indices in male adults with obesity as a function of regular exercise. Exp Physiol. (2019) 104:1650–60. 10.1113/EP087862 31609518

[47] Faul F Erdfelder E Buchner A Lang AG. Statistical power analyses using G^*^ Power 3.1: tests for correlation and regression analyses. Behav Res Method. (2009) 41:1149–60. 10.3758/BRM.41.4.1149 19897823

[48] Cohen J. A power primer. Psychol Bull. (1992) 112:155–9. 10.1037/0033-2909.112.1.155 19565683

[49] Hansen CJ Stevens LC Coast JR. Exercise duration and mood state: how much is enough to feel better?. Health Psychol. (2001) 20:267. 10.1037/0278-6133.20.4.267 11515738

[50] Vogt T Herpers R Scherfgen D Strüder HK Schneider S. Neuroelectric adaptations to cognitive processing in virtual environments: an exercise-related approach. Exp Brain Res. (2015) 233:1321–9. 10.1007/s00221-015-4208-x 25630906

[51] Grahn P Stigsdotter UK. The relation between perceived sensory dimensions of urban green space and stress restoration. Landsc Urban Plan. (2010) 94:264–75. 10.1016/j.landurbplan.2009.10.012

[52] Spitzer RL Kroenke K Williams JB Löwe B. A brief measure for assessing generalized anxiety disorder: the GAD-7. Arch Intern Med. (2006) 166:1092–7. 10.1001/archinte.166.10.1092 16717171

[53] Kroenke K Spitzer RL Williams JB Monahan PO Löwe B. Anxiety disorders in primary care: prevalence, impairment, comorbidity, and detection. Ann Intern Med. (2007) 146:317–25. 10.7326/0003-4819-146-5-200703060-00004 17339617

[54] Lee SH Shin C Kim H Jeon SW Yoon HK Ko YH. Validation of the Korean version of the generalized anxiety disorder 7 self-rating scale. Asia Pacific Psychiatry. (2022) 14:e12421. 10.1111/appy.12421 32893471

[55] Omani-Samani R Maroufizadeh S Ghaheri A Navid B. Generalized anxiety Disorder-7 (GAD-7) in people with infertility: a reliability and validity study. Middle East Fertil Soc J. (2018) 23:446–9. 10.1016/j.mefs.2018.01.01331500644

[56] Tiirikainen K Haravuori H Ranta K Kaltiala-Heino R Marttunen M. Psychometric properties of the 7-item Generalized Anxiety Disorder Scale (GAD-7) in a large representative sample of Finnish adolescents. Psychiatry Res. (2019) 272:30–5. 10.1016/j.psychres.2018.12.004 30579178

[57] Zhang C Wang T Zeng P Zhao M Zhang G Zhai S. Reliability, validity, and measurement invariance of the general anxiety disorder scale among chinese medical university students. Front Psychiatry. (2021) 12:648755. 10.3389/fpsyt.2021.648755 34093269 PMC8170102

[58] Noone BM Robson SK (2016). Understanding consumers' inferences from price and nonprice information in the online lodging purchase decision. Serv Sci. 8:108–23. 10.1287/serv.2016.0141

[59] Wedel M Pieters R. Visual marketing: From attention to action. Psychology Press. (2012).

[60] Atalay AS Bodur HO Rasolofoarison D. Shining in the center: Central gaze cascade effect on product choice. J Cons Res. (2012) 39:848–66. 10.1086/665984

[61] Wang TC Tsai CL Tang T.W. Exploring advertising effectiveness of tourist hotels' marketing images containing nature and performing arts: An eye-tracking analysis. Sustainability. (2018) 10:3038. 10.3390/su10093038

[62] Manor BR Gordon E. Defining the temporal threshold for ocular fixation in free-viewing visuocognitive tasks. J Neurosci Method. (2003) 128:85–93. 10.1016/S0165-0270(03)00151-1 12948551

[63] Nikolaev AR Jurica P Nakatani C Plomp G van Leeuwen C. Visual encoding and fixation target selection in free viewing: Presaccadic brain potentials. Front Sys Neurosci. (2013) 7:26. 10.3389/fnsys.2013.00026 23818877 PMC3694272

[64] Wang Y Sparks BA An An eye-tracking study of tourism photo stimuli: image characteristics and ethnicity. J Trav Res. (2016) 55:588–602. 10.1177/0047287514564598

[65] Loyola P Martinez G Muñoz K Velásquez JD Maldonado P Couve A. Combining eye tracking and pupillary dilation analysis to identify Website Key Objects. Neurocomputing. (2015) 168:179–89. 10.1016/j.neucom.2015.05.108

[66] Nie B Huang X Chen Y Li A Zhang R Huang J. Experimental study on visual detection for fatigue of fixed-position staff. Appl Ergon. (2017) 65:1–11. 10.1016/j.apergo.2017.05.010 28802427

[67] Browning M Behrens TE Jocham G O'reilly JX Bishop SJ. Anxious individuals have difficulty learning the causal statistics of aversive environments. Nat Neurosci. (2015) 18:590. 10.1038/nn.3961 25730669 PMC4644067

[68] Dindia K Allen M. Sex differences in self-disclosure: a meta-analysis. Psychol Bull. (1992) 112:106. 10.1037/0033-2909.112.1.106 1388280

[69] Shanahan DF Franco L Lin BB Gaston KJ Fuller RA. The benefits of natural environments for physical activity. Sports Med. (2016) 46:989–95. 10.1007/s40279-016-0502-4 26886475

[70] Goldberg JH Kotval XP. Computer interface evaluation using eye movements: methods and constructs. Int J Ind Ergon. (1999) 24:631–45. 10.1016/S0169-8141(98)00068-7

[71] Calvo MG Lang PJ. Gaze patterns when looking at emotional pictures: Motivationally biased attention. Motiv Emot. (2004) 28:221–43. 10.1023/B:MOEM.0000040153.26156.ed

[72] Leder H Mitrovic A Goller J. How beauty determines gaze! Facial attractiveness and gaze duration in images of real world scenes. Iperception. (2016) 7:2041669516664355. 10.1177/2041669516664355 27698984 PMC5030765

[73] Lang PJ Greenwald MK Bradley MM Hamm AO. Looking at pictures: affective, facial, visceral, and behavioral reactions. Psychophysiology. (1993) 30:261–73. 10.1111/j.1469-8986.1993.tb03352.x 8497555

[74] Franěk M Šefara D PetruŽálek J Cabal J Myška K. Differences in eye movements while viewing images with various levels of restorativeness. J Environ Psychol. (2018) 57:10–6. 10.1016/j.jenvp.2018.05.001

[75] Gavas R Chatterjee D Sinha A. Estimation of cognitive load based on the pupil size dilation. in 2017 IEEE International Conference on Systems, Man, and Cybernetics (SMC). Banff, AB (2017). p. 1499–504. 10.1109/SMC.2017.8122826

[76] Herzog TR Rector AE. Perceived danger and judged likelihood of restoration. Environ Behav. (2008) 41:387–401. 10.1177/0013916508315351

[77] Tabrizian P Baran PK Smith WR Meentemeyer RK. Exploring perceived restoration potential of urban green enclosure through immersive virtual environments. J Environ Psychol. (2018) 55:99–109. 10.1016/j.jenvp.2018.01.001

[78] Gatersleben B Andrews M. When walking in nature is not restorative—the role of prospect and refuge. Health Place. (2013) 20:91–101. 10.1016/j.healthplace.2013.01.001 23399852

